# Analysis of neurodegenerative disease-causing genes in dementia with Lewy bodies

**DOI:** 10.1186/s40478-020-0879-z

**Published:** 2020-01-29

**Authors:** Tatiana Orme, Dena Hernandez, Owen A. Ross, Celia Kun-Rodrigues, Lee Darwent, Claire E. Shepherd, Laura Parkkinen, Olaf Ansorge, Lorraine Clark, Lawrence S. Honig, Karen Marder, Afina Lemstra, Ekaterina Rogaeva, Peter St. George-Hyslop, Elisabet Londos, Henrik Zetterberg, Kevin Morgan, Claire Troakes, Safa Al-Sarraj, Tammaryn Lashley, Janice Holton, Yaroslau Compta, Vivianna Van Deerlin, John Q. Trojanowski, Geidy E. Serrano, Thomas G. Beach, Suzanne Lesage, Douglas Galasko, Eliezer Masliah, Isabel Santana, Pau Pastor, Pentti J. Tienari, Liisa Myllykangas, Minna Oinas, Tamas Revesz, Andrew Lees, Brad F. Boeve, Ronald C. Petersen, Tanis J. Ferman, Valentina Escott-Price, Neill Graff-Radford, Nigel J. Cairns, John C. Morris, Stuart Pickering-Brown, David Mann, Glenda Halliday, David J. Stone, Dennis W. Dickson, John Hardy, Andrew Singleton, Rita Guerreiro, Jose Bras

**Affiliations:** 1 UK Dementia Research Institute at UCL and Department of Molecular Neuroscience, Institute of Neurology, University College London, London, UK; 2 Laboratory of Neurogenetics, National Institutes on Aging, NIH, Bethesda, MD USA; 3 Department of Neuroscience, Mayo Clinic, Jacksonville, FL USA; 4 Center for Neurodegenerative Science, Van Andel Research Institute, Grand Rapids, Michigan USA; 5 Neuroscience Research Australia, Sydney, Australia and School of Medical Sciences, Faculty of Medicine, University of New South Wales, Sydney, Australia; 6 Nuffield Department of Clinical Neurosciences, Oxford Parkinson’s Disease Centre, University of Oxford, Oxford, UK; 7 Taub Institute for Alzheimer Disease and the Aging Brain and Department of Pathology and Cell Biology and Neurology, Columbia University Medical Center, New York, NY USA; 8 Department of Neurology and Alzheimer Center, Neuroscience Campus Amsterdam, VU University Medical Center, Amsterdam, the Netherlands; 9 Department of Medicine, Tanz Centre for Research in Neurodegenerative Diseases, University of Toronto, Toronto, ON Canada; 10 Clinical Memory Research Unit, Institution of Clinical Sciences Malmö, Lund University, Lund, Sweden; 11 UK Dementia Research Institute at UCL and Department of Molecular Neuroscience, Institute of Neurology, University College London, London, UK; 12 Clinical Neurochemistry Laboratory, Institute of Neuroscience and Physiology, Sahlgrenska Academy at the University of Gothenburg, Mölndal, Sweden; 13 Human Genetics, School of Life Sciences, University of Nottingham, Nottingham, UK; 14 Department of Basic and Clinical Neuroscience and Institute of Psychiatry, Psychology and Neuroscience, King’s College London, London, UK; 15 Queen Square Brain Bank, Department of Molecular Neuroscience, UCL Institute of Neurology, London, UK; 16 Parkinson’s Disease & Movement Disorders Unit, Neurology Service, Clinical Neuroscience Institute (ICN), Hospital Clínic, Institut de Neurociències, University Universitat de Barcelona, IDIBAPS, Barcelona, Catalonia Spain; 17 Department of Pathology and Laboratory Medicine, Center for Neurodegenerative Disease Research, Perelman School of Medicine at the University of Pennsylvania, 3600 Spruce Street, Philadelphia, USA; 18 Banner Sun Health Research Institute, 10515 W Santa Fe Drive, Sun City, AZ 85351 USA; 19 Sorbonne Université, Université Pierre et Marie Curie-Paris 06, Inserm, Centre National de la Reserche Scientifique, and Institute du Cerveau et de la Moelle épinière, Paris, France; 20 Assistance Publique Hôpitaux de Paris, Hôpital de la Salpêtrière, Département de Génétique et Cytogénétique, Paris, France; 21 Division of Neurosciences and Laboratory of Neurogenetics, National Institute on Aging/NIH, Bethesda, MD USA; 22 Neurology Service, University of Coimbra Hospital, Coimbra, Portugal; 23 Memory Unit, Department of Neurology, University Hospital Mutua de Terrassa, University of Barcelona, Terrassa, Barcelona, Spain; 24 Centro de Investigación Biomédica en Red Enfermedades Neurodegenerativas (CIBERNED), Madrid, Spain; 25 Translational Immunology, Research Programs Unit, Department of Neurology, Helsinki University Hospital, University of Helsinki, Helsinki, Finland; 26 Department of Pathology, University of Helsinki and HUSLAB, Helsinki University Hospital, Helsinki, Finland; 27 Department of Neuropathology and Neurosurgery, Helsinki University Hospital and University of Helsinki, Helsinki, Finland; 28 Neurology Department, Mayo Clinic, Rochester, MN USA; 29 Department of Psychiatry and Department of Psychology, Mayo Clinic, Jacksonville, FL USA; 30 UK Dementia Research Institute at Cardiff and MRC Centre for Neuropsychiatric Genetics and Genomics, School of Medicine, Cardiff University, Cardiff, UK; 31 Department of Neurology, Mayo Clinic, Jacksonville, FL USA; 32 Department of Neurology, Washington University School of Medicine, Saint Louis, MO USA; 33 Division of Neuroscience and Experimental Psychology, Faculty of Biology, Medicine and Health, University of Manchester, Manchester, UK; 34 Genetics and Pharmacogenomics, Merck & Co., Inc., West Point, PA USA

## Abstract

Dementia with Lewy bodies (DLB) is a clinically heterogeneous disorder with a substantial burden on healthcare. Despite this, the genetic basis of the disorder is not well defined and its boundaries with other neurodegenerative diseases are unclear. Here, we performed whole exome sequencing of a cohort of 1118 Caucasian DLB patients, and focused on genes causative of monogenic neurodegenerative diseases. We analyzed variants in 60 genes implicated in DLB, Alzheimer’s disease, Parkinson’s disease, frontotemporal dementia, and atypical parkinsonian or dementia disorders, in order to determine their frequency in DLB. We focused on variants that have previously been reported as pathogenic, and also describe variants reported as pathogenic which remain of unknown clinical significance, as well as variants associated with strong risk. Rare missense variants of unknown significance were found in *APP, CHCHD2, DCTN1, GRN, MAPT, NOTCH3, SQSTM1, TBK1* and *TIA1*. Additionally, we identified a pathogenic *GRN* p.Arg493* mutation, potentially adding to the diversity of phenotypes associated with this mutation. The rarity of previously reported pathogenic mutations in this cohort suggests that the genetic overlap of other neurodegenerative diseases with DLB is not substantial. Since it is now clear that genetics plays a role in DLB, these data suggest that other genetic loci play a role in this disease.

## Introduction

Dementia with Lewy bodies (DLB) is a neurodegenerative disease that shares clinical and pathological features with both Parkinson’s disease (PD) and Alzheimer’s disease (AD). A disease most often occurring in the elderly demographic, it exhibits a varied clinical presentation that can include dementia, parkinsonism, visual hallucinations, REM sleep behavior disorder, fluctuations in attention and alertness, as well as autonomic and psychiatric dysfunction [49], all described as clinical features of underlying synucleinopathy. Similar to PD, the pathological hallmark of DLB is accumulation of alpha-synuclein in Lewy bodies (LBs) and Lewy neurites, leading to consideration of these conditions as different ends of the same clinico-pathological spectrum. Lewy-related pathology in DLB can be found not only in the brainstem, but also widespread in limbic and/or neocortical regions [47]. Alzheimer pathology in the form of amyloid plaques and tau neurofibrillary tangles are frequently found at autopsy [17].

At present, knowledge of the genetic etiology of DLB is limited. Families with the disorder are rare, and *SNCA* multiplications and point mutations have been shown to cause disease in multiplex families of mixed Parkinson’s disease and dementia [31, 63, 77]. Variants in *PRNP* [38] and *SNCB* [55] have been reported in DLB, but their pathogenicity has been questioned due to lack of replication, lack of segregation [55], or presence in healthy controls [8]. DLB shares risk loci that are associated with AD or PD, and we have recently shown the genetic correlation between DLB and PD, and DLB and AD, is approximately equal when disregarding *APOE* [26]. The *APOE* ε4 allele strongly predisposes to the development of the disorder [13, 57, 64, 69], as do certain variants in the *GBA* gene [53]. A non-synonymous variant in *PLCG2* has been proposed to confer protection from AD [62], and more recently, DLB and frontotemporal dementia (FTD) [71]. An association at the *SNCA* locus was identified, which, interestingly, shows a differential association profile in DLB than that seen in PD [13]. Furthermore, the first genome-wide association study (GWAS) was recently published, analyzing the genotyping data from 1743 DLB patients, a proportion of whom (745 samples) are also included in this study. In a two-stage study design, the associations at *APOE, GBA* and *SNCA* were replicated, and novel loci reached suggestive levels of association [27]. It has been proposed that rare variants in AD and PD causing genes may also play a role in sporadic DLB. However, these studies have been small, with sample sizes of approximately 100 cases [36, 37, 51]; it is thus uncertain whether these findings are merely coincidental.

We performed exome sequencing of over 1000 DLB patients to investigate the role of genetic variants in not only AD and PD genes, but additionally in a collection of approximately 40 disease genes established as causative of neurodegenerative diseases. The genetic variants studied were those previously implicated in FTD with or without amyotrophic lateral sclerosis (ALS); atypical parkinsonian disorders; and disorders with dementia as a presenting feature.

As DLB is a heterogeneous disorder, and has overlapping features with other diseases, accurate diagnosis relies on the combination of clinical and pathological assessments. To mitigate this issue as much as possible, the DLB patients included in this study had a neuropathological diagnosis of DLB.

## Methods

### Cohort studied

We studied 1118 patients neuropathologically diagnosed according to the 2005 McKeith diagnosis guidelines [49], as meeting the criteria for ‘intermediate’ or ‘high’ likelihood DLB and self-reported as Caucasian. The mean age at death in the cohort was 78.8 (±8.4) years and the male to female ratio was 1.5.

### Exome sequencing and data analysis

DNA was extracted from the cerebellum or frontal cortex using standard methods [75]. Exome sequencing was performed using Illumina’s Nextera Rapid Expanded Exome capture (62 Mb), or Agilent SureSelect Exome Capture Kit (v4) and sequenced on Illumina’s HiSeq2500 using 100 bp paired-end reads. On target average coverage of at least 30x was obtained for all included samples. Data analysis was performed according to standard GATK (v3) best practices [50] using a single informatics pipeline and performing joint variant calling of single nucleotide variants (SNVs) and short insertions and deletions (indels) across all samples. In brief, sequencing reads were aligned to the human reference genome (GRCh37/hg19) using bwa-mem (v0.7.12), duplicate reads were flagged using samblaster (v0.1.21), and realignment around indels and base quality scores were recalibrated using GATK. Variant recalibration was performed using GATK’s variant quality score recalibration (VQSR) [21, 70]. Variants that did not meet the VQSR threshold of 99.9 were excluded. Individual genotypes with a phred-scaled quality score below 20 and with coverage below 8 were set to missing. Variants were included only if they had a high call rate in both capture kits (genotyped in ≥90% of samples). Annotation of variants was performed with snpEff (v4.2) [15] and dbNSFP v2.9 [44] using GRCh37/hg19 as reference.

### Sample quality control (QC)

Sample quality control metrics were generated using PLINK 1.9 [58]. Population structure was analysed using principal component analysis, and samples that did not cluster with the European population of the 1000 Genomes dataset were removed from analysis. Concordance between reported and genotype sex was performed for each sample to remove those with a discordant sex assignment. Samples with inconsistent heterozygosity rates, or that were shown to be related or duplicated were excluded. Due to linkage disequilibrium, population structure and genotyping error, we removed an individual from a pair of samples using a proportion of identity by descent threshold of ≥0.1875 [3]. After QC measures, we analyzed variants from 1004 DLB patients. The locations from which the DLB samples were sourced can be found in Additional file 1: Table S1.

### Neurodegenerative disease genes

Fifty-seven genes were selected based on their role in monogenic forms of neurodegenerative diseases including AD, PD, FTD and related Mendelian disorders featuring parkinsonism or dementia. Due to its relevance to DLB, we also report on variants in *GBA* and *APOE*, as well as the *PLCG2* p.Pro522Arg variant. A full list of the studied genes can be found in Additional file 2: Table S2.

We focused on variants that were amino acid changing; or that fell in splice donor/acceptor sites; in splice regions, or in 5′ or 3′ untranslated regions.

A literature search was conducted for each disease-causing gene studied in order to identify previously reported pathogenic mutations. This included primary literature reports; supplementary information; the Human Gene Mutation Database [66]; the Online Mendelian Inheritance in Man website [2]; the AD & FTD, and PD mutation databases [20]; and Clinvar [39]. Population variant frequency was determined using gnomAD (v2.1), a genomic database consisting of variants from 125,748 exomes and 15,708 genomes [34, 41].

The maximum frequency that a known pathogenic variant occurs in a large cohort of a disease of interest allows for an estimation of the maximum tolerated frequency of a pathogenic variant for that disease [74]. As the genetics of DLB is yet to be fully delineated, we used Alzheimer’s disease to estimate a maximum tolerated allele count for a pathogenic variant to occur in the gnomAD database, implemented using the alleleFrequencyApp http://cardiodb.org/allelefrequencyapp/ [74]. This was used as a conservative approximation of maximum tolerated variant frequencies for pathogenic mutations in the general population for a disease such as AD.

Reported variants were also analysed for their frequency in 432 control individuals from the Healthy Exomes (HEX) dataset, who died aged 60 or over, without any disease-associated neuropathology [29, 28].

### *APOE* genotyping

Given the difficulty in sequencing the *APOE* locus, the *APOE* haplotype was confirmed in samples where DNA was available (*n* = 758 samples). *APOE* haplotype genotyping was conducted using enzyme restriction, as previously described [29].

### Sanger sequencing

Candidate variants were confirmed by Sanger sequencing when DNA was available. DNA was amplified by polymerase chain reaction using Roche FastStart PCR Master Mix (Roche Diagnostics Corp) and sequenced with Applied Biosystems BigDye terminator version 3.1 sequencing chemistry in an ABI3730XL genetic analyzer as per manufacturer’s instructions (Applied Biosystems). Primers are available upon request. The sequences were analysed using Sequencher software version 4.2 (Gene Codes).

## Results

We performed a detailed analysis of the genetic variability of 57 genes shown previously to cause neurodegenerative diseases in over 1000 DLB cases. Furthermore, we also report on *GBA* and *APOE,* well established risk genes for DLB, as well as the recently reported protective *PLCG2* p.Pro522Arg variant.

We identified a pathogenic nonsense mutation in *GRN*, p.Arg493* (ENST00000053867), in one patient. This variant was confirmed by Sanger sequencing. In addition, we identified previously reported mutations that, due to their low frequency in the general population, have uncertain clinical significance (Table 1). No previously reported pathogenic homozygous variants were identified in genes that cause neurodegenerative disease with autosomal recessive inheritance. As the data was not phased, compound heterozygous variants could not be completely assessed.

**Table 1:** Heterozygous variants of uncertain clinical significance that were found in the DLB case cohort. The variant in *DCTN1* was found in two DLB cases. All other variants were seen in single DLB cases. Variants are included in Table 1 if they were previously reported, but not fully established as pathogenic, and if they were present five times or less in the European (non-Finnish) population in gnomAD

Gene	Transcript	Variant	Number of DLB patients	GnomAD MAF (all populations)	GnomAD NFE AC (Total NFE AN)	Previously reported	Additional information	Ref
*APP*	ENST00000346798	c.2020G > A p.Glu674Lys	1	0	Not present	In AD: adjacent amino acid (p.A673T) protective, or (p.A673V) causes EOAD when homozygous	Variant newly identified here. Adjacent amino acid plays a role in disease	[22, 32]
*CHCHD2*	ENST00000395422	c.10G > A p.Gly4Arg	1	0.00002840	4 (128432)	1 LBD patient	–	[54]
*DCTN1*	ENST00000361874	c.2339 T > C p.Ile780Thr	2	0.00003889	5 (129150)	1 patient with FALS, control	Low frequency in gnomAD, but also found in a control	[35]
*GRN*	ENST00000053867	c.827C > T p.Ala276Val	1	0.000007075	1 (129048)	1 patient with an initial diagnosis of FTD which was changed to depression	Variant found in a patient included in a neurodegenerative disease cohort, but diagnosis was changed from FTD	[76]
*MAPT*	ENST00000344290	c.256G > A p.Gly86Ser	1	0.00002901	4 (126300)	1 patient with clinical FTD	Segregation not proven	[65]
*NOTCH3*	ENST00000263388	c.1732C > T, p.Arg578Cys	1	0.00003989	2 (113234)	Found in several CADASIL, but also seen in 1 control	Homozygous variant carrier had a mild phenotype	[14, 33, 43, 68]
		c.1733G > A p.Arg578His	1	0.000007980	0 (113200)	p.Arg578Cys (different amino acid change) seen in CADASIL	Different amino acid change than previously reported, not cysteine changing	[14]
		c.1820G > A p.Arg607His	1	0.000004000	1 (112826)	1 CADASIL, and a different amino acid change, p.Arg607Cys seen in several CADASIL patients	Filtered site in gnomAD, may be false positive. Not cysteine changing	[1, 46, 67]
*SQSTM1*	ENST00000389805	c.1210A > G p.Met404Val	1	0.00001591	3 (113732)	Several families with Paget’s disease of bone	Variant shown to cause Paget’s disease of bone (not FTD-ALS)	[23, 30]
*TBK1*	ENST00000331710	c.1150C > T p.Arg384Trp	1	0.00002128	4 (128892)	p.Arg384Thr (different amino acid change) reported in 1 ALS	Different amino acid change than previously reported. Variation in the same amino acid in 2 different DLB cases in this study	[12]
		c.1151G > A p.Arg384Gln	1	0.00008514	3 (128842)	p.Arg384Thr (different amino acid change) reported in 1 ALS	Different amino acid change than previously reported. Variation in the same amino acid in 2 different DLB cases in this study	[12]
*TIA1*	ENST00000433529	c.1085C > T p.Pro362Leu	1	0.00002121	2 (129146)	2 s degree relatives with FTD/ALS	Unreplicated report suggests this variant causes ALS/FTD	[45]

*Ref* Reference, *MAF* Minor Allele Frequency, *gnomAD NFE* Non-Finnish European, *AC* Allele Count, *AN* Allele Number, *FTD/ALS* Frontotemporal Dementia / Amyotrophic Lateral Sclerosis, *CADASIL* Cerebral Autosomal Dominant Arteriopathy with Subcortical Infarcts and Leukoencephalopathy, *EOAD* Early Onset Alzheimer’s disease, *FALS* Familial Amyotrophic Lateral Sclerosis, *SALS* Sporadic Amyotrophic Lateral Sclerosis, *LBD* Lewy Body Disease, *AD* Alzheimer’s disease, *PD* Parkinson’s disease

### *GRN* p.Arg493*

The patient with the p.Arg493* *GRN* mutation had a medical history that included a stroke, and previous surgical evacuation of a subdural haematoma, as well as a family history of AD in a parent and a sibling, with onset in the seventh decade. The presenting symptom was episodes of confusion, and a CT scan showed moderate cerebral and cerebellar atrophy with a lacunar infarct in the left external capsule. Subsequent symptoms included memory loss, disorientation, altered gait, stooped posture, right-sided rigidity and visual hallucinations. Impaired smooth pursuit, left hemiparesis, left spasticity, right-sided rigidity, and a slight parkinsonian tremor of the left hand were also reported. The patient was severely demented and scored 3/30 on the Mini Mental State Examination (MMSE) in an assessment conducted at 83 years, approximately 2 years before death.

Macroscopically, the right convexity of the brain showed very marked asymmetrical atrophy of the frontal, temporal and parietal lobes, with knife-edge atrophy at the frontal and temporal poles, and mild atrophy of the right occipital lobe. There were no focal lesions. Pathological examination with immunohistochemical staining for alpha-synuclein protein showed a single LB in the substantia nigra; very infrequent Lewy bodies in multiple cortical areas; sparse LBs in the anterior cingulate, entorhinal and transentorhinal cortices; and no LBs in the amygdala. Thioflavin S methods showed frequent diffuse amyloid plaques in neocortical areas. Very infrequent neurofibrillary tangles were present in the nucleus basalis of Meynert, hypothalamus and entorhinal cortex, and were absent from all neocortical areas examined. Immunohistochemical staining for abnormal tau protein identified frequent dot-like features and diffusely stained neurons in the entorhinal cortex, transentorhinal cortex, CA1 and subiculum of the hippocampus and the parahippocampal gyrus. There were sparse argyrophilic grains in the amygdala, entorhinal cortex and area CA1 of the subiculum. Immunohistochemical staining for ubiquitin showed moderate densities of short neuropil threads and dots, as well as sparse to moderate densities of small cytoplasmic inclusions in layer II of frontal and temporal neocortical regions. Following the identification of the mutation, the sample was stained for TDP-43 and revealed inclusions in frontal and temporal lobes. Sections stained immunohistochemically for phosphorylated TDP-43 protein showed immunoreactive dystrophic puncta, neurites and perikaryal cytoplasmic inclusions within layer II of frontal and temporal neocortical areas, as well as the amygdala, hippocampal CA1 region, and entorhinal cortex. These features reached moderate to frequent densities in the frontal and temporal neocortex.

### Established risk factors for DLB

No homozygous Gaucher disease causing variants were identified in *GBA*. Table 2 details variants found in *GBA* that had a call-rate of 90% or greater. Two variants, p.Glu365Lys and p.Asn409Ser were over-represented in DLB compared to the gnomAD database. Of note, 2 homozygous carriers of p.Glu365Lys were identified and a novel variant, p.Asn409del, was identified in 1 patient in the cohort.

**Table 2:** Variants in *GBA* that had a call-rate of 90% or over in the DLB cohort

Variant		Annotation	Heterozygous DLB cases	DLB MAF	MAF in- house controls (*n* = 432)	gnomAD NFE AF	gnomAD NFE AC, AN (number of homozygotes)
1:155205518 C/G (rs1064651)	p.Asp448His	missense variant	2	0.000991	0	0.000179, failed quality filters	AC = 23, AN = 128,640 (0)
1:155205563 C/A (rs80356769)	p.Val433Leu	missense variant	2	0.000991	0	0	AC = 0, AN = 113,740 (0)
1:155205581 C/T (rs149171124)	p.Glu427Lys	missense variant	2	0.000991	0	0.000263	AC = 34, AN = 129,176 (0)
1:155205632 GGTT/G	p.Asn409del	inframe deletion & splice region variant	1	0.000547	0	0	NA
1:155205634 T/C (rs76763715)	p.Asn409Ser	missense variant & splice region variant	19	0.009958	0.0023202	0.002045	AC = 264, AN = 129,124 (2)
1:155205638 A/G (rs377143075)	c.1225-3 T > C	splice region variant & intron variant	1	0.000529	0	0.000093	AC = 12, AN = 129,160 (0)
1:155206117 A/G (rs755021234)	p.Cys381Cys	synonymous variant	1	0.000496	0	0.000009	AC = 1, AN = 113,740 (0)
1:155206157 C/T (rs1064648)	p.Arg368His	missense variant	1	0.000496	0	0.000101	AC = 13, AN = 129,174 (0)
**1:155206167 C/T (rs2230288)**	**p.Glu365Lys**	**missense variant**	**75**	**0.039187**	**0.01042**	**0.012340**	**AC = 1594, AN = 129,170 (11)**
1:155206187 G/A	p.Pro358Leu	missense variant	1	0.000496	0	0	NA
1:155206200 C/G (rs398123526)	p.Asp354His	missense variant	1	0.000496	0	0.000026	AC = 3, AN = 113,764 (0)
1:155206262 T/G	c.1000-2A > C	splice acceptor variant & intron variant	1	0.000496	0	0.000000	NA
1:155207965 C/T (rs409652)	p.Gly241Arg	missense variant	2	0.000992	0	0.000035	AC = 4, AN = 113,766 (0)
1:155208060 C/G	p.Arg209Pro	missense variant	1	0.000496	0	0	NA
1:155209539 G/A (rs368145008)	p.Leu108Leu	synonymous variant	1	0.000520	0	0.000023	AC = 3, AN = 129,172 (0)
1:155209737 G/A (rs1141812)	p.Arg83Cys	missense variant	2	0.000992	0	0.000054	AC = 7, AN = 129,080 (0)
1:155210918 T/C (rs41264927)	c.-15A > G	5 prime UTR premature start codon gain variant	2	0.000991	0	0.001641	AC = 212, AN = 129,174 (0)

All variants were found in the heterozygous state, apart from the variant in bold, p.Glu365Lys, which was found as a homozygous variant in 2 DLB cases. The frequency of these variants are also reported in 432 in-house controls who died aged 60 or over without disease neuropathology. *MAF* Minor Allele Frequency, *gnomAD NFE* Non-Finnish European, *AC* Allele Count, *AN* Allele Number

*APOE* haplotypes were independently genotyped in 758 DLB samples where DNA was available. Of this subset of samples, the ε4 allele frequency was 48.6%, in keeping with the prevalence of this allele in previous reports [64, 69].

### Potentially pathogenic variants

We used Alzheimer’s disease genetics as a model in order to apply an approximate threshold for the tolerated occurrence of pathogenic AD mutations in gnomAD. We applied an AD prevalence of 1/79 (Alzheimer Society Dementia report, 2014); a maximum estimated disease-causing MAF of 0.0012; and a very conservative penetrance threshold of 0.5. The maximum tolerated frequency for a pathogenic allele for AD using the allele number of a variant genotyped in the entire gnomAD European (non-Finnish) population was 1.5556 × 10^− 5^, or five alleles. This is a very conservative approximation, likely to be too high since AD is more prevalent than DLB, however since the genetic architecture of DLB still remains largely unresolved, we used AD as an approximation. In Table 1 we describe previously reported variants of unknown clinical significance that were present in 5 or less Europeans in the gnomAD database. None of these variants were detected in the control individuals from the HEX dataset. These variants were identified in *APP, CHCHD2, DCTN1, GRN, MAPT, NOTCH3, SQSTM1, TBK1* and *TIA1.* Variants were reported in Table 1 if the evidence for pathogenicity was moderate, such as having unproven segregation with disease; eliciting a different amino acid change than was previously reported; identified in a gene that has not been replicated as causative for disease; or if the variant has also been identified in a control subject. Table 1 also shows variants not present in Europeans in gnomAD that affect the same or an adjacent amino acid as previously reported in disease. Table 3 lists variants identified in multiple DLB cases and with 5 or less alleles in gnomAD.

**Table 3:** Variants found in more than 1 DLB case, that were present in 5 or less individuals from the gnomAD exome dataset of non-Finnish Europeans

Gene	Transcript	Variant	Number of DLB cases	GnomAD NFE AC (Total NFE AN)
*PARK2*	ENST00000366898	p.Gln440Arg	2	1 (112822)
*VPS13C*	ENST00000261517	p.Ile288Met	2	4 (112094)
*TUBA4A*	ENST00000248437	p.Val181Met	2	5 (113764)
*DNAJC6*	ENST00000371069	p.Val632Ala	2	0 (83222)
*VPS35*	ENST00000299138	p.Arg499His	2	4 (113646)

All variants were identified in the heterozygous state. *gnomAD NFE* Non-Finnish European, *AC* Allele Count, *AN* Allele Number

Variants that have previously been reported in disease, but were more frequent in the general population, thus less likely to be fully penetrant, disease-causing alleles, are reported in Additional file 3: Table S3. These include variants such as *SNCA* p.His50Gln, which is present in 19 Europeans in gnomAD, and whose pathogenicity has been disputed [10].

### Genes previously reported in DLB

We investigated variants in genes that have been reported as causative of DLB - *PRNP* and *SNCB* - as well as a gene in which a variant was recently reported as protective for DLB - *PLCG2*.

The previously identified *PRNP* p.Met232Arg [38] variant was not present in our cohort, and there were no other previously reported pathogenic variants in *PRNP* in our data. It is therefore unlikely that *PRNP* mutations cause sporadic DLB in the European population.

The *SNCB* p.Val70Met and p.Pro123His variants reported to predispose to DLB [55] were also not found in our cohort of DLB cases, suggesting that if they play a role in DLB, they may be population specific risk factors. In our cohort, no non-synonymous variants were found in the *SNCB* gene, even though the entire gene was adequately covered.

A variant in *PLCG2,* p.Pro522Arg, has been reported to reduce the risk of DLB, AD and FTD [71]. This variant was identified in 18 DLB cases (MAF 0.0089), which is similar to the frequency found in gnomAD non-Finnish Europeans (MAF 0.0087).

### TREM2

*TREM2* is the second strongest genetic risk factor for AD, an effect largely driven by the p.R47H variant. In our data, the p.Arg47His variant, which was successfully sequenced in 667 of the 1004 samples, had a MAF 0.00299, which is similar to the frequency in NFEs in gnomAD (MAF 0.002466). The p.Arg62His variant also had a similar MAF in DLB (0.0142), compared to Europeans in the gnomAD database (0.0112). Likewise, the frequency of p.Thr96Lys and p.Leu211Pro variants was similar in DLB patients and gnomAD, (MAF 0.000991 versus 0.00101, and MAF 0.000992 versus 0.00113), respectively. No homozygous variants in *TREM2* were identified.

## Discussion

We present a comprehensive analysis of rare genetic variability in an extensive number of neurodegenerative disease-causing genes in a large cohort of patients diagnosed with DLB. We used state-of-the-art analytical approaches with well-established quality control criteria that allowed us to identify genetic variability and estimate its contribution to disease.

Mutations in *GRN* cause FTD [7, 19], and the most commonly reported pathogenic mutation in *GRN* is p.Arg493* [59]. The patient described here lacked prominent clinical signs of FTD, such as changes in behavior, personality, or language impairment. Severe dementia, parkinsonism and visual hallucinations were present, and led to the suggested clinical diagnosis of Alzheimer’s disease or mixed vascular dementia, with a final neuropathological diagnosis of Alzheimer’s disease, dementia with Lewy bodies and argyrophilic grain disease. Substantial phenotypic variability has been described in patients with a *GRN* p.Arg493* mutation. In 34 patients identified with this mutation, 25 had a diagnosis of FTD; 4 of primary progressive aphasia; 3 of corticobasal syndrome and 3 of Alzheimer’s disease. Age at onset ranged from 44 to 69, and the most common initial symptom was a change in personality or executive dysfunction (25/33 patients), with subsequent language impairment occurring in 26 of 33 patients. Fourteen out of thirty patients had parkinsonism and 10% had visual hallucinations [59]. This mutation has also been found in 4 patients with sporadic Alzheimer’s disease [24].

An overlap between FTD and DLB has been shown both clinically and neuropathologically. Several patients have been identified that simultaneously met clinical criteria for both FTD and DLB [16, 52], or that presented clinically with FTD, but at autopsy had DLB [11, 61]*.* Concomitant TDP-43 and alpha-synuclein pathology can be found in 27–60% of DLB cases at autopsy [4, 17, 48]. In general, *GRN* mutations result in disease with clinical heterogeneity [40, 72, 73], and it is possible that overlaps between DLB and FTD may be connected to variation in *GRN.* It is also possible that hallucinations and delusions in *GRN* carriers may cause a misdiagnosis of DLB [9]. An FTD case harboring the *GRN* p.Thr382fs mutation showed a phenotype resembling DLB, with fluctuations in cognition, parkinsonism and visual hallucinations [5]. Furthermore, alpha-synuclein and TDP-43 pathologies were found in a subset of brains of patients with a *GRN* mutation [42]. A small study in 58 DLB cases showed that together, rare variants in *GRN* are associated with DLB [37], although this is yet to be independently replicated. A recent study identified 9 heterogeneous FTLD cases with coexisting Lewy body pathology Braak stage ≥ IV, comprising 7% of the cohort. Two of the 9 cases had a secondary diagnosis of DLB, and a further 2 had *GRN* mutations [25]. Progranulin and β-amyloid have been shown to co-localize in plaques in DLB, suggesting a possible biological association between these two aggregated proteins [60]. However, there are often multiple concomitant pathologies that are identified in neurodegenerative disease, and so the observation of TDP-43 with Lewy and amyloid pathology could simply be coincidental multi-morbidity of simultaneous pathologies that coexist in the ageing brain [6]. After the identification of the *GRN* mutation, additional clinicopathological information was reviewed to assess this case. The histopathological findings of the p.Arg493* carrier revealed Lewy bodies; amyloid plaques; argyrophilic grains; ubiquitin positive inclusions; tau staining, and when reanalysed, TDP-43 pathology. The medical history reported the occurrence of a stroke, which was also confirmed by a CT scan, however no further clinical information was available. Taken together, these facts add to the difficulty in classifying these more complex forms of disease. Particularly in archive cases where the neuropathology assessment did not originally include staining for TDP-43.

We identified possible pathogenic variants in other genes linked to the FTD-ALS spectrum of disease. A variant in *TIA1*, p.Pro362Leu, suggested to be causative of ALS/FTD, was found in 1 DLB patient in the cohort. *TIA1* encodes an RNA-binding protein, and this variant was reported in a pair of second degree relatives with ALS/FTD, as well as a clinically symptomatic, but non-demented relative. An increased burden of rare heterozygous *TIA1* mutations in a larger ALS/FTD cohort was reported [45]. In two neuropathologically diagnosed DLB cases, we found two variants in *TBK1* that affected the same amino acid: p.Arg384Trp and p.Arg384Gln. This amino acid has been found to be altered in a sporadic Sardinian ALS case (p.Arg384Thr) [12]: a variant not present in gnomAD, but whose pathogenicity is unconfirmed. It is intriguing that two DLB cases were identified with variants that affect the same amino acid. These variants are present in gnomAD (MAFs 0.0000213 and 0.0000851, respectively). At present, their contribution to DLB is unclear.

From the perspective of AD related genetics, we identified a variant of unknown significance in *APP* - p.Glu674Lys - in a DLB case, a substitution of a negatively charged amino acid to a positively charged amino acid at the 3rd residue of the ß-amyloid peptide. This variant was not present in the gnomAD database, and affects an adjacent amino acid to that previously described. The p.Ala673Val was shown to cause early onset Alzheimer’s disease in the homozygous state, whereas heterozygous individuals in the family were unaffected [22]. A different amino acid change at the same position, p.Ala673Thr, has been reported in a patient without AD [56], and predicted as non pathogenic due to non segregation with disease in a family [18]. Furthermore, this variant was shown to be protective against Alzheimer’s disease in the Icelandic population, where it resulted in a reduction of beta-secretase cleavage in vitro [32].

We have also looked at strong risk modulating genes: *GBA*, *TREM2, APOE* and *PLCG2*. If at *GBA* and *APOE* we saw evidence of increased frequency in the previously reported variants, we did not observe such a finding at *TREM2* or *PLCG2*, with both showing frequencies that are identical to those found in the general population.

We have also identified a number of possible pathogenic mutations in several other genes studied here (Table 1). The design of this study does not allow variant pathogenicity to be unequivocally established, and, in this way, we report these variants to allow future studies to attempt confirmation of these findings.

In summary, we provide the first large-scale characterization of rare genetic variability in the most relevant neurodegenerative disease-causing genes in DLB. Our findings suggest that mutations in genes known to cause other neurodegenerative diseases are not a common cause of DLB.

## Supplementary information

**Additional file 1: Table S1.** Sources of samples. Research groups, clinical teams and brain banks where the DLB samples included in this study were collected from.

**Additional file 2: Table S2.** Neurodegenerative disease-causing genes and DLB risk genes analysed in this study. Genes known to cause neurodegenerative diseases are presented according to the mode of inheritance of the respective mendelian disease. Genes such as *PARK2*, *FBXO7*, *SYNJ1*, and *DNAJC6*, among others, are commonly referred to as parkinson’s disease genes, although the clinical and pathological characteristics may be atypical in some cases. FTD/ALS - frontotemporal dementia/Amyotrophic lateral sclerosis, CADASIL - Cerebral arteriopathy, autosomal dominant, with subcortical infarcts and leukoencephalopathy, CARASIL - Cerebral arteriopathy, autosomal recessive, with subcortical infarcts and leukoencephalopathy *Both *TMEM230* and *DNAJC13* have been hypothesised to be the cause of Parkinson’s disease in the same family.

**Additional file 3: Table S3.** Variants identified in the studied DLB cohort that have been previously reported in disease and have a gnomAD european allele count > 5. GnomAD NFE AC = gnomAD non-Finnish European allele count. GnomAD NFE AN = gnomAD non-Finnish European allele number. GnomAD Total MAF = gnomAD all populations minor allele frequency.
