# Supplementary material for: Analysis of neurodegenerative disease-causing genes in dementia with Lewy bodies
Source: Acta Neuropathol Commun. 2020 Jan 29;8:5. doi: 10.1186/s40478-020-0879-z (PMC6990558; doi:10.1186/s40478-020-0879-z)
Supplement: Supplementary file 1 — Additional file 1: Table S1. Sources of samples. Research groups, clinical teams and brain banks where the DLB samples included in this study were collected from. [file 40478_2020_879_MOESM1_ESM.docx]

Supplementary table 1: Sources of samples. Research groups, clinical teams and brain banks where the DLB samples included in this study were collected from.

| **Samples’ providers** | **Number of samples** |
| --- | --- |
| University of New South Wales, Australia | 76 |
| University of Bristol, UK | 45 |
| University of Toronto, Canada | 2 |
| Banner Sun Health, USA | 17 |
| Mayo Clinic, USA | 164 |
| University College London, UK | 17 |
| University of Helsinki, Finland | 16 |
| University Pierre and Marie Curie, France | 14 |
| Harvard Brain Bank, USA | 68 |
| Kings College London, UK | 71 |
| University of Malmo, Sweden | 43 |
| University of Manchester, UK | 42 |
| The Netherlands Brain Bank | 132 |
| University of Nottingham, UK | 13 |
| Columbia University, New York, USA | 56 |
| University of Oxford, UK | 61 |
| Hospital de la Santa Creu i Sant Pau, Barcelona, Spain | 13 |
| University of California San Diego, USA | 12 |
| University of Pennsylvania, USA | 6 |
| University of Washington St. Louis, USA | 136 |
| **Total** | **1004** |
