# Supplementary material for: Analysis of neurodegenerative disease-causing genes in dementia with Lewy bodies
Source: Acta Neuropathol Commun. 2020 Jan 29;8:5. doi: 10.1186/s40478-020-0879-z (PMC6990558; doi:10.1186/s40478-020-0879-z)
Supplement: Supplementary file 2 — Additional file 2: Table S2. Neurodegenerative disease-causing genes and DLB risk genes analysed in this study. Genes known to cause neurodegenerative diseases are presented according to the mode of inheritance of the respective mendelian disease. Genes such as PARK2, FBXO7, SYNJ1, and DNAJC6, among others, are commonly referred to as parkinson’s disease genes, although the clinical and pathological characteristics may be atypical in some cases. FTD/ALS - frontotemporal dementia/Amyotrophic lateral sclerosis, CADASIL - Cerebral arteriopathy, autosomal dominant, with subcortical infarcts and leukoencephalopathy, CARASIL - Cerebral arteriopathy, autosomal recessive, with subcortical infarcts and leukoencephalopathy *Both TMEM230 and DNAJC13 have been hypothesised to be the cause of Parkinson’s disease in the same family. [file 40478_2020_879_MOESM2_ESM.docx]

Supplementary table 2: Neurodegenerative disease-causing genes and DLB risk genes analysed in this study.

| Mode of inheritance | Gene | Disease(s) |
| --- | --- | --- |
| Autosomal Dominant Inheritance | *APP* | Alzheimer's disease |
|  | *ATP1A3* | Rapid onset dystonia parkinsonism, others |
|  | *CCNF* | FTD/ALS |
|  | *CHCHD10* | FTD/ALS |
|  | *CHCHD2* | Parkinson's disease |
|  | *CHMP2B* | Frontotemporal Dementia |
|  | *COL4A1* | Hereditary multi-infarct dementia (Swedish) |
|  | *CSF1R* | Hereditary diffuse leukoencephalopathy with spheroids |
|  | *CYLD* | FTD/ALS |
|  | *DCTN1* | Perry syndrome, atypical features |
|  | *DNAJC13** | Parkinson's disease |
|  | *DNMT1* | Hereditary Sensory Neuropathy Type IE |
|  | *FUS* | FTD/ALS |
|  | *GCH1* | Dopa responsive dystonia |
|  | *GRN* | Frontotemporal Dementia |
|  | *HNRNPA1* | FTD/ALS |
|  | *HNRNPA2B1* | FTD/ALS |
|  | *ITM2B* | British Dementia |
|  | *LRRK2* | Parkinson's disease |
|  | *MAPT* | Frontotemporal Dementia |
|  | *MATR3* | ALS |
|  | *NOTCH3* | CADASIL |
|  | *PRKAR1B* | FTD |
|  | *PRNP* | Creutzfeldt-Jakob disease, others |
|  | *PSEN1* | Alzheimer's disease |
|  | *PSEN2* | Alzheimer's disease |
|  | *SERPINI1* | Familial encephalopathy with neuroserpin inclusion bodies |
|  | *SNCA* | Parkinson's disease |
|  | *SNCB* | DLB |
|  | *SQSTM1* | FTD/ALS |
|  | *TARDBP* | FTD/ALS |
|  | *TBK1* | FTD/ALS |
|  | *TIA1* | FTD/ALS |
|  | *TMEM230** | Parkinson’s disease |
|  | *TUBA4A* | ALS +- FTD |
|  | *VCP* | FTD/ALS |
|  | *VPS35* | Parkinson's disease |
| Autosomal Recessive inheritance | *ATP13A2* | Kufor-rakeb disease, Parkinson's disease |
|  | *DNAJC6* | Early onset Parkinson's disease |
|  | *FBXO7* | Early onset Parkinson's disease |
|  | *HTRA1* | CARASIL |
|  | *OPTN* | FTD ALS |
|  | *PANK2* | Pantothenate kinase-associated neurodegeneration (PKAN) or neurodegeneration with brain iron accumulation |
|  | *PARK2* | Early onset Parkinson's disease |
|  | *PARK7* | Early onset Parkinson's disease |
|  | *PINK1* | Early onset Parkinson's disease |
|  | *PLA2G6* | Parkinson's disease, Phospholipase A2-associated neurodegeneration (PLAN) |
|  | *SPG11* | Juvenile ALS, spastic paraplegia and charcot-marie tooth disease |
|  | *SYNJ1* | Early onset Parkinson's disease |
|  | *TH* | Segawa syndrome |
|  | *TREM2* | Frontotemporal Dementia, polycystic lipomembranous osteodysplasia with sclerosing leukoencephalopathy |
|  | *TYROBP* | Polycystic lipomembranous osteodysplasia with sclerosing leukoencephalopathy |
|  | *VPS13C* | Early onset Parkinson’s disease |
| Autosomal dominant or Recessive inheritance | *POLG* | Progressive external ophthalmoplegia and parkinsonism |
| X-linked | *ATP6AP2* | X-linked parkinsonism with spasticity |
|  | *RAB39B* | Waisman Syndrome |
|  | *UBQLN2* | FTD/ALS |
| DLB risk genes | *GBA* | |
|  | *APOE* | |
|  | *PLCG2* | |

Genes known to cause neurodegenerative diseases are presented according to the mode of inheritance of the respective mendelian disease. Genes such as *PARK2*, *FBXO7*, *SYNJ1*, and *DNAJC6*, among others, are commonly referred to as Parkinson’s disease genes, although the clinical and pathological characteristics may be atypical in some cases. FTD/ALS - Frontotemporal dementia/Amyotrophic lateral sclerosis, CADASIL - Cerebral arteriopathy, autosomal dominant, with subcortical infarcts and leukoencephalopathy, CARASIL - Cerebral arteriopathy, autosomal recessive, with subcortical infarcts and leukoencephalopathy *Both *TMEM230* and *DNAJC13* have been hypothesised to be the cause of Parkinson’s disease in the same family.
