# Supplementary material for: Analysis of neurodegenerative disease-causing genes in dementia with Lewy bodies
Source: Acta Neuropathol Commun. 2020 Jan 29;8:5. doi: 10.1186/s40478-020-0879-z (PMC6990558; doi:10.1186/s40478-020-0879-z)
Supplement: Supplementary file 3 — Additional file 3: Table S3. Variants identified in the studied DLB cohort that have been previously reported in disease and have a gnomAD european allele count > 5. GnomAD NFE AC = gnomAD non-Finnish European allele count. GnomAD NFE AN = gnomAD non-Finnish European allele number. GnomAD Total MAF = gnomAD all populations minor allele frequency. [file 40478_2020_879_MOESM3_ESM.docx]

Supplementary table 3: Variants identified in the studied DLB cohort that have been previously reported in disease and have a gnomAD european allele count >5. GnomAD NFE AC = gnomAD non-Finnish European allele count. GnomAD NFE AN = gnomAD non-Finnish European allele number. GnomAD Total MAF = gnomAD all populations minor allele frequency.

| Gene | Transcript | Variant DNA | Variant protein | GnomAD NFE AC | GnomAD NFE AN | GnomAD Total MAF |
| --- | --- | --- | --- | --- | --- | --- |
| *APP* | ENST00000346798 | c.*18C>T | NA | 6 | 126668 | 0.00002526 |
| *PSEN1* | ENST00000324501 | c.1309A>G | p.Ile437Val | 6 | 126716 | 0.00002164 |
| *TBK1* | ENST00000331710 | c.452C>T | p.Ser151Phe | 6 | 113330 | 0.00002396 |
| *SQSTM1* | ENST00000389805 | c.332C>T | p.Pro111Leu | 11 | 126454 | 0.00003982 |
| *TYROBP* | ENST00000585901 | c.140T>C | p.Val47Ala | 12 | 126380 | 0.0000759 |
| *VCP* | ENST00000358901 | c.79A>G | p.Ile27Val | 12 | 126724 | 0.0006529 |
| *PSEN2* | ENST00000366782 | c.514G>A | p.Val172Met | 13 | 126600 | 0.0001083 |
| *LRRK2* | ENST00000298910 | c.2769G>C | p.Gln923His | 16 | 126166 | 0.0001701 |
| *SNCA* | ENST00000336904 | c.150T>G | p.His50Gln | 19 | 126694 | 0.00007937 |
| *GCH1* | ENST00000395514 | c.610G>A | p.Val204Ile | 20 | 126496 | 0.0001949 |
| *NOTCH3* | ENST00000263388 | c.850G>A | p.Ala284Thr | 23 | 124638 | 0.0002816 |
| *FUS* | ENST00000568685 | c.*48G>A | NA | 28 | 126514 | 0.00013 |
| *COL4A1* | ENST00000375820 | c.3946C>G | p.Gln1316Glu | 29 | 126700 | 0.0001263 |
| *FUS* | ENST00000568685 | c.170_172delCTT | p.Ser57del | 31 | 126734 | 0.0001731 |
| *TBK1* | ENST00000331710 | c.871A>G | p.Lys291Glu | 33 | 125810 | 0.0001348 |
| *POLG* | ENST00000268124 | c.3436C>T | p.Arg1146Cys | 36 | 126616 | 0.000184 |
| *CHMP2B* | ENST00000263780 | c.85A>G | p.Ile29Val | 37 | 126542 | 0.0001624 |
| *LRRK2* | ENST00000298910 | c.7067C>T | p.Thr2356Ile | 39 | 126416 | 0.0001772 |
| *NOTCH3* | ENST00000263388 | c.2932A>C | p.Ser978Arg | 40 | 110588 | 0.0002439 |
| *TBK1* | ENST00000331710 | c.964C>T | p.His322Tyr | 40 | 122924 | 0.0004478 |
| *PSEN2* | ENST00000366782 | c.283C>T | p.Arg95Cys | 41 | 126472 | 0.000213 |
| *FUS* | ENST00000568685 | c.*132C>A | NA | 42 | 84388 | 0.0002179 |
| *POLG* | ENST00000268124 | c.2542G>A | p.Gly848Ser | 43 | 126688 | 0.0001768 |
| *POLG* | ENST00000268124 | c.2207A>G | p.Asn736Ser | 52 | 126646 | 0.00035 |
| *LRRK2* | ENST00000298910 | c.5606T>C | p.Met1869Thr | 54 | 126586 | 0.0003321 |
| *TARDBP* | ENST00000240185 | c.269C>T | p.Ala90Val | 57 | 126710 | 0.0002164 |
| *LRRK2* | ENST00000298910 | c.4111A>G | p.Ile1371Val | 69 | 126258 | 0.0008638 |
| *GRN* | ENST00000053867 | c.359C>A | p.Ser120Tyr | 75 | 126654 | 0.001036 |
| *LRRK2* | ENST00000298910 | c.2378G>T | p.Arg793Met | 130 | 25792 | 0.001061 |
| *PSEN2* | ENST00000366782 | c.488C>T | p.Ser163Leu | 142 | 126562 | 0.0006357 |
| *LRRK2* | ENST00000298910 | c.356T>C | p.Leu119Pro | 276 | 126528 | 0.001235 |
| *CHCHD2* | ENST00000395422 | c.5C>T | p.Pro2Leu | 339 | 126242 | 0.01011 |
| *DNAJC13* | ENST00000260818 | c.4547G>A | p.Arg1516His | 405 | 126058 | 0.002076 |
| *DNAJC13* | ENST00000260818 | c.6509T>G | p.Leu2170Trp | 541 | 126496 | 0.002459 |
